# Growth in healthy infants with cow's milk protein allergy fed extensively hydrolyzed or amino acid-based formulas

**DOI:** 10.1186/s40795-024-00901-6

**Published:** 2024-07-19

**Authors:** Armen Malekiantaghi, Mahbobeh Aghajani, Hosein Shabani-Mirzaee, Mohsen Vigeh, Kambiz Eftekhari

**Affiliations:** 1 Department of Pediatric Gastroenterology, Bahrami Children’s Hospital, School of Medicine, Tehran University of Medical Sciences, Tehran, Iran; 2 Department of Pediatric, Bahrami Children’s Hospital, School of Medicine, Tehran University of Medical Sciences, Tehran, Iran; 3 Department of Pediatric Endocrinology, Bahrami Children’s Hospital, School of Medicine, Tehran University of Medical Sciences, Tehran, Iran; 4 Maternal-Fetal Medicine Research Center, Tehran University of Medical Sciences, Tehran, Iran; 5 Pediatric Gastroenterology and Hepatology Research Center, Bahrami Children’s Hospital, Tehran University of Medical Sciences, Tehran, Iran

**Keywords:** Allergy to cow’s milk protein, Amino acid-based formula, Extensively hydrolyzed formula, Infant, Growth

## Abstract

**Background:**

Cow’s milk protein allergy (CMPA) is one of the most common food allergies in the first year of life. Special formulas for infants with CMPA include extensively hydrolyzed (EHFs) and amino acid-based (AAFs) formulas. The aim of this study was to evaluate the growth of infants fed with these special formulas.

**Methods:**

This was a prospective chart review study that evaluated the growth criteria of infants with CMPA fed with EHFs or AAFs until one year of age. These infants were referred to the gastroenterology clinic of Bahrami Children's Hospital from April 2021 to April 2022. These infants were divided into two groups, the group fed with EHFs and AAFs. Then growth criteria were evaluated in both groups.

**Results:**

Fifty-eight children were enrolled in the study, of which 51.7% were girls. Forty were consuming the EHFs formulas. The median time of both diagnosis and treatment was 60 days. The most common clinical manifestations were gastroesophageal reflux, dysentery, eczema, vomiting, and cough, respectively. The diagnosis of the disease in the AAFs group was significantly earlier than in the other group. The growth of children in both groups was completely proportional to their age and growth criteria at birth. Comparing the groups, all growth parameters were higher in the EHFs group.

**Conclusion:**

This study showed that the growth criteria (weight, length, and head circumference) were suitable for each group and were in accordance with the WHO growth charts compared to the birth criteria. But in the group fed with EHFs, compared to AAFs, the growth rate was higher.

**Supplementary Information:**

The online version contains supplementary material available at 10.1186/s40795-024-00901-6.

## Background

Cow's milk protein allergy (CMPA) is one of the most common food allergies and is known as the most common food allergy in the first year of life [1]. CMPA occurs in about 7% of formula-fed and 0.5% of breast-fed infants. Early initiation of cow's milk formula, especially in the first three days of life, is associated with the occurrence of CMPA [2]. The clinical manifestations of this disease include skin symptoms (rash and eczema), digestive symptoms (nausea and vomiting and abdominal pain), and respiratory symptoms (runny nose and wheezing). Allergy to cow's milk protein can cause reaction by two mechanisms dependent and independent of immunoglobulin E (IgE). In the non-dependent type (non-IgE allergy), the reaction is delayed and occurs hours or days after contact and can be accompanied by skin, digestive or respiratory manifestations and the best diagnostic method is the challenge test [3]. In the dependent type (IgE allergy), the reaction is usually rapid and occur within two hours after consuming milk and can be accompanied by skin rashes and vomiting [4]. This type of allergy is diagnosed by skin test or blood test [5]. In recent decades, the prevalence, persistence and severity of cow's milk protein allergy have increased [6]. The European Society of Pediatric Gastroenterology, Hepatology and Nutrition (ESPGHAN) and the American Academy of Pediatrics (AAP) recommend extensively hydrolyzed (EHFs) and amino acid-based (AAFs) formulas for feeding children with CMPA [7]. The first choice is a EHFs formula. AAFs should be chosen in the following cases: there is no improvement within 2–4 weeks after the start of EHFs, symptoms are very severe or there are multiple food allergies [8]. The protein content and ingredients of these two types of formulas are similar, but the EHFs formula contains only free amino acids and is inherently non-allergenic [9]. According to Sova et al.'s study, children with food allergies are at greater risk for growth disorders and inadequate nutrient intake [10]. Some studies have shown impaired growth in these infants [11, 12], and conversely some other studies have shown that food allergy has no effect on growth, even in children with multiple food allergies [13–15]. On the other hand, Mennella et al.'s study also showed that the growth of infants who are fed with EHF and AAF formulas is different from the growth of infants who are fed with other formula or breast milk [16]. In general, limited studies have been conducted on the long-term effects of these formulas on the growth of infants [17]. Therefore, considering the different opinions about the presence or absence of growth disorders in these infants, we decided to evaluate the effects of feeding with hypoallergenic formulas (EHFs compared to AAFs formula) on growth parameters’ (weight, length and head circumference) of infants with CMPA up to one year old.

## Methods

### Study design

This was a prospective chart review study. Infants with CMPA referred to the gastroenterology clinic of Bahrami Children's Hospital for one year, from April 2021 to April 2022, whose disease was previously diagnosed based on clinical symptoms (vomiting, gastroesophageal reflux, bloody diarrhea…) and family history, were confirmed by a pediatric gastroenterologist and feeding with hypoallergenic formulas and supplementary feeding without allergens had been started. Inclusion criteria including formula-fed infants younger than 1 year old who were on a hypoallergenic diet with a definitive diagnosis of CMPA and had referred to the gastroenterology outpatient clinic of Bahrami Children's Hospital. Infants with prematurity, anaphylaxis caused by allergy to the cow's milk protein, eosinophilic gastrointestinal disorders, chronic systemic diseases, celiac disease, cystic fibrosis, congenital metabolic diseases, chronic infections, Immunodeficiency, and malignancy were not included in the study (as the exclusion criteria). The information of this study was collected by the researcher and colleagues of the project. For this, a questionnaire containing demographic information was designed (Supplementary Table). It was completed by interviewing the parents of infants on the first visit to the clinic. Infants' growth parameters were obtained from birth until the time of referral to our clinic using information recorded in the medical record and vaccine card. Post-referral, these parameters were measured and recorded during periodic follow-ups. The evaluation periods for these parameters included birth, two, four, six, and twelve months. In the case of previous visits, their medical records were observed and additional information was extracted from them. Then the weight, length, and head circumference of the infants were measured and recorded in their questionnaire. The type of sampling in this study was “simple non-random sampling”. In the one-year period from April 2021 to April 2022, all eligible samples that were referred to the gastroenterology outpatient clinic of Bahrami Children's Hospital were included in the study. At first, the objectives of the study were explained to the parents of the eligible infants, and written informed consent was obtained from them if they were willing to enroll in the study. The diagnosis of CMPA was previously given by a pediatric gastroenterologist, based on clinical manifestations and family history, and feeding was started with a special formula and food without any cow’s proteins. All these infants were formula-fed and based on the CMPA protocol, they were first fed with EHFs formula and in the severe cases or lack of response after 2 weeks of initiation EHFs, and they were given a AAFs formula. Patients were divided into two groups based on the type of hypoallergenic formula: EHFs formulas and AAFs formulas. In both groups, in addition to the special formula, the hypoallergenic diet was also followed. A separate questionnaire was prepared for each infant. Demographic information including gender, time of disease diagnosis, time of starting special feeding, type of hypoallergenic formula (EHFs or AAFs formula), pregnancy status, and mother's education level were recorded. Then weight, length, and head circumference were measured and recorded according to the standard method. Other information related to growth criteria from the time of birth (including time of birth, two, four, and six months) was obtained from the vaccine card or the previous medical record and recorded in their questionnaire. An infant weighing scale and length meter was used to measure weight and length respectively. Also, use an infant head circumference measuring tape to measure the head circumference. Finally, the rate of growth of each infant during one year and his/her final growth at the end of one year were compared with herself. The growth status of each group was evaluated at birth, two, four, six months, and one year, and finally, the growth status of the two groups was compared.

### Ethical considerations

The patients voluntarily entered the study after obtaining written informed consent from their parents that the patient's information remains confidential with the researcher. Patients could withdraw from the study at any time. In this study, the ethical principles and provisions of the Helsinki Convention were observed. This study was approved by the ethics committee of Tehran University of Medical Sciences and the ethics code IR.TUMS.CHMC.REC.1400.050 was assigned to it.

### Statistical analysis

The obtained information was recorded in Microsoft Excel software. For the final analysis was used SPSS software version 24. The distribution of quantitative variables (age, weight, length, and head circumference) of children was not normal in the Kolmogorov–Smirnov test, so the non-parametric Mann–Whitney U test was used to evaluate the differences between the two groups. Number and percentage were used to express qualitative variables such as sex, birth order, and mother's education. Chi-square test was used to check the statistical difference of qualitative variables between groups. Mean, median, and standard deviation were used to display quantitative variables, and frequency and percentage were used to display qualitative variables. The ANCOVA test were used to eliminate potential confounding factors. Statistically, values less than 0.05 were considered significant.

## Results

During the study, seventy-four children with a previous diagnosis of CMPA were referred to the gastroenterology outpatient clinic of Bahrami Children’s Hospital. Sixteen infants were excluded from the study due to a history of prematurity or underlying diseases (e.g., hypothyroidism), and finally, fifty-eight children were enrolled and completed the study. There were 30 girls (51.7%) and 28 boys (48.3%) in total. Among them, 40 infants were fed with EHFs formula, while 18 were fed with AAFs formula. Throughout the study, no changes in milk consumption were observed within the groups. Additionally, all of the infants started consuming cow's protein-free supplemental food after reaching 4 months of age. The median time to diagnosis of the disease in all samples was 60 days (interquartile range: 30–90), the shortest recorded time was 10 days and the longest was 150 days. The median time of starting special feeding in all samples was 60 days (interquartile range: 30–90), the earliest recorded time was 15 days and the latest time was 270 days. The median time interval between the diagnosis of the disease and the start of special feeding in all samples was zero days (interquartile range: 0–30), the lowest interval was zero days (the start of special feeding at the same time the diagnosis of the disease) and the longest interval was 240 days. The most common initial clinical manifestations of the patients that led to the diagnosis of CMPA and the initiation of special feeding were gastroesophageal reflux (47%), dysentery (24%), eczema (19%), vomiting (5%) and cough (5%), respectively. In this study, the education level of the mother; 45% (*n* = 26) had a diploma, 4% (*n* = 2) associate degree, 41% (*n* = 24) bachelor's and 10% (*n* = 6) master’s.

The summary of the demographic characteristics of the studied infants, separated into two groups, was reported in Table 1.

**Table 1:** Demographic findings of patients separated into two groups fed with extensively hydrolyzed (EHFs) and amino acid based (AAFs) formulas

Variables		EHFs	AAFs	*P*-Value
Gender	Female	24 (60%)	6 (33.3%)	^‡^0.85
	Male	16 (40%)	12 (66.7%)	
Birth order	First	24 (60%)	14 (77.8%)	^**‡**^** < 0.0001**
	Second	14 (35%)	4 (22.2%)	
	Third	2 (5%)	0 (0.0%)	
Mother’s education	Diploma	14 (35%)	12 (66.7%)	^**‡**^**0.001**
	Associate degree	2 (5%)	0 (0%)	
	Bachelor’s degree	18 (45%)	6 (33.3%)	
	Master’s degree	6 (15%)	0 (0.0%)	
Time of diagnosis (days)		60 (30–90)	30 (22.5–45)	**0.02**^**†**^
Start time of special feeding (days)		90 (60–120)	30 (30–60)	**0.01**^**†**^
The interval between diagnosis and the start of special feeding (days)		15 (0–30)	0 (0–22.5)	0.21^**†**^

Findings are reported as number (percentage) and median (interquartile range)

*EHFs* Extensively hydrolyzed formulas, *AAFs* Amino Acid Based Formulas

^‡^Chi-square test

^†^Mann-Whitty test

Infant’s growth criteria were among the most important variables measured in this study. Table 2 summarizes the changes in the infant’s growth criteria, including weight, length, head circumference, and body mass index separated into two groups, from birth to one year of age.

**Table 2:** Comparison of the growth criteria of the studied infants, separated into two groups fed with extensively hydrolyzed (EHFs) and amino acid based (AAFs) formulas

Variables		EHFs	AAFs	*P*-Value^a^	*P*-Value^c^
Weight	At birth	3292 (200)	3232 (221)	0.31^b^	
(gram)	Two months	5000 (362)	4605 (374)	**0.001**	
	Four months	6005 (357)	5700 (302)	**0.001 > **	
	Six months	7055 (486)	6811 (370)	**0.028**	
	One year	9220 (804)	8811 (509)	**0.02**^b^	**0.08**
Weight gain	During the first year	5928 (738)	5578 (493)	0.09^**c**^	0.76
Height	At birth	50 (1.06)	49.56 (0.98)	0.13	
(centimeter)	Two months	57.35 (0.97)	56.22 (0.64)	**0.001 > **	
	Four months	63.30 (1.01)	62.00 (1.37)	**0.001**	
	Six months	68.00 (1.28)	66.89 (1.13)	**0.002**	
	One year	75.85 (1.54)	74.44 (1.09)	**0.001**	**0.001**
Height increase	During the first year	25.85 (1.54)	24.8 (1.13)	**0.003**^**c**^	**0.02**
Head circumference	At birth	34.60 (1.25)	33.56 (0.85)	**0.002**	
(centimeter)	Two months	38.58 (1.12)	37.67 (0.68)	**0.001 > **	
	Four months	41.50 (1.30)	40.56 (1.29)	**0.012**	
	Six months	43.55 (1.17)	43.11 (0.90)	0.098	
	One year	45.80 (1.18)	45.11 (1.02)	**0.023**	**0.27**
Head circumference Increase	During the first year	11.20 (0.88)	11.55 (0.85)	0.87^**c**^	0.36
BMI	At birth	13.27 (0.87)	13.15 (0.56)	0.057	
	Two months	15.27 (0.93)	14.68 (1.08)	0.046	
	Four months	15.20 (0.89)	14.98 (1.06)	0.71	
	Six months	15.36 (1.31)	15.33 (0.54)	0.34	
	One year	16.13 (1.46)	16.43 (1.01)	0.91	0.4
BMI Increase	During the first year	2.85 (1.24)	3.27 (1.22)	0.27^**c**^	0.34
Weight for height percentile	At birth	39.23 (21.57)	41.89 (4.68)	0.46	
	Two months	34.48 (21.14)	28.89 (18.66)	0.33	
	Four months	13.85 (12.33)	11.67 (7.77)	0.49	
	Six months	17.58 (17.20)	5.89 (2.8)	**0.001 > **	
	One year	33.15 (23.64)	12.0 (11.53)	**0.001 > **	**0.002**
Weight for height percentile change	During the first year	-6.07 (27.04)	-29.88(10.84	**0.001 > **	0.001

Findings are reported as number (percentage) and median (interquartile range)

*EHFs* Extensively hydrolyzed formulas, *AAFs* Amino Acid Based Formulas, *BMI* Body Mass Index

^a^Mann-Whitney test

^b^Independent t test

^c^ANCOVA test (growth criteria at birth, mother’s education level & birth order adjusted)

The frequency of boys was higher in the AAFs group, but this difference was not significant. The frequency of the first child in the two groups is higher than in other birth orders, but the ratio of this frequency is significantly higher in the group of children fed with AAFs formulas. There was a statistically significant difference between the two groups in terms of the level of the mother's education so the diploma education was more in the AAFs group and the bachelor's education was more in the EHFs group. After controlling for the influence of maternal education and birth order, there was no significant difference in weight and head circumference at 12 months (0.08 and 0.27, respectively). However, there were statistically significant differences in length and weight-for-length percentile at 12 months (0.02 and 0.001, respectively). The diagnosis of the disease and, as a result, the start of special feeding in the AAFs group was significantly earlier than in the EHFs group (one month versus two months). The median interval between the diagnosis of the disease and the start of special feeding was zero day in the AAFs group and 15 days in the EHFs group, but this difference was not statistically significant.

According to the WHO growth charts (weight for age, length for age, and head circumference for age), the growth of children in both groups was completely proportional to their age and birth criteria, and no growth disorders were reported during the first year of life (Table 2).

**Fig. 1:**
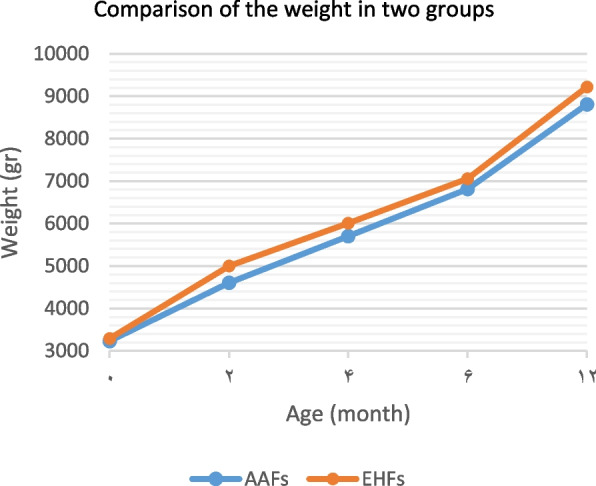
Comparison of the weight in two groups of infants fed with extensively hydrolyzed (EHFs) and amino acid based (AAFs) formulas

**Fig. 2:**
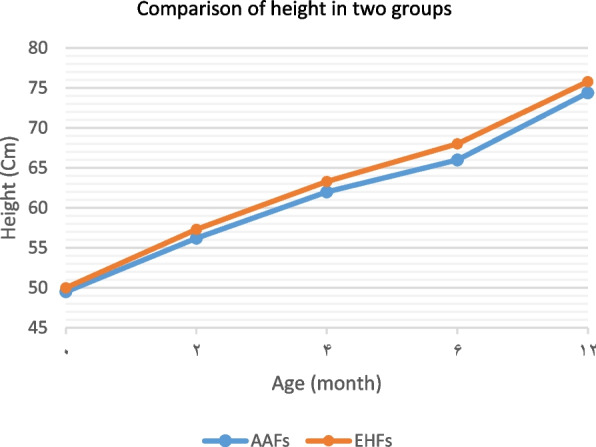
Comparison of length in two groups of infants fed with extensively hydrolyzed (EHFs) and amino acid based (AAFs) formulas

**Fig. 3:**
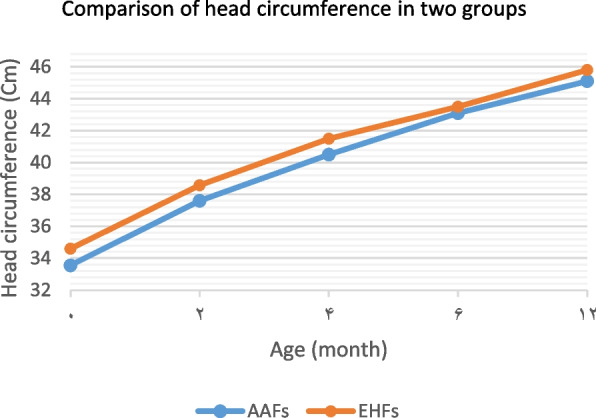
Comparison of head circumference in two groups of infants fed with extensively hydrolyzed (EHFs) and amino acid based (AAFs) formulas

**Fig. 4:**
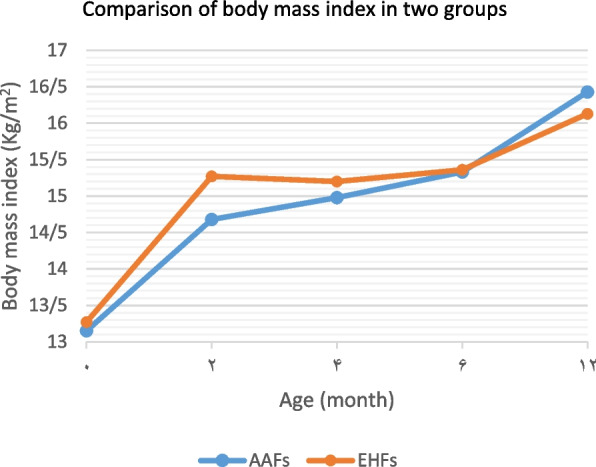
Comparison of body mass index in two groups of infants fed with extensively hydrolyzed (EHFs) and amino acid based (AAFs) formulas

In comparing the growth criteria between the two groups, in all the age periods of the first year, the mean weight, head circumference, and body mass index were higher in the EHFs group than that the AAFs group. But these differences were not significant at the end of one year. The mean length was also higher in the EHFs group than in the AAFs group in all the age periods of the first year. This difference was significant at the end of one year (Table 2). The Comparing of infants’ growth criteria in the first year of life in the groups was shown in Figs. 1, 2, 3 and 4.

## Discussion

The main aim of this study was to evaluate the growth rate of infants with CMPA, under a hypoallergenic diet and feeding with EHFs or AAFs formula, and to compare the growth criteria in these two groups. The results of the present study showed that the growth criteria in both groups were proportional to the infant's age and in accordance with the WHO growth charts and within the normal range. On the other hand, all growth parameters were higher in infants fed with EHFs formula compared to AAFs formula. But at the end of the first year, there was a significant difference only in length increase.

A study by Canani et al. showed that feeding with AAFs formula was safe and allowed adequate growth in infants with CMPA [18]. In another study, it has been stated that EHFs and AAFs are special formulas and formulated to feed infants suffering from CMPA, but they are nutritionally complete formulas and meet the nutritional and growth needs of this group of children [19]. Vandenplas et al.'s study also shows that feeding with AAFs formulas, along with a complementary diet without cow's protein, supports normal growth up to 9 months of age [17]. These results were consistent with our study. Other studies, like our, indicate the absence of growth disorders in children with food allergies and fed with special formulas, even in children with multiple food allergies [13–15].

Meyer et al.’s study found that dietary restrictions for managing food allergies increased the risk of growth disorders in these children [20]. Some studies have also shown that avoiding cow's milk may negatively affect the growth of infants and young children [11, 21]. Burks also believed that children with cow's milk allergy were at risk of inadequate nutrition and growth [22]. A study by Sova et al. also found that children with multiple food allergies are at greater risk for growth impairment and inadequate nutrient intake compared to children without food allergies [10]. A number of studies also suggest that food allergy in general [13, 23, 24] and CMPA in particular are potential risk factors for lower weight-for-age percentiles, length-for-age percentiles, or both [23, 25, 26]. The results of these studies are contrary to ours. In our study, growth disorders were not seen in any of the groups consuming hypoallergenic formulas.

Borschel et al.’s study reported that the growth differences of infants fed with EHFs and AAFs are mostly related to the type of formula and the composition of protein, fat and bioavailability of the basic nutrients in them [19]. While Maslin believed that feeding problems and refusal to feed are very common problems in infants with CMPA, which can negatively affect their nutrition and dietary intake [27]. In our study, it was also seen that growth in both groups was proportional to age, but in the group fed with AAFs, the growth criteria were significantly lower than in the EHFs group, which can be due to the following reasons: The presence of multiple food allergies, severe digestive disorders secondary to chronic allergic inflammation including gastroesophageal reflux disease (GERD), increased metabolic needs, accompanying other allergic diseases such as concomitant asthma or atopic dermatitis. In these cases, the findings of Mehta et al.'s study were similar to ours [28]. A study by Niggemann et al. showed that infants fed EHFs had significantly less vomiting and more frequent soft stools than infants fed AAFs [29]. This can also be the reason for better growth in the group fed with EHFs, as in our study patients.

In the present study, the diagnosis of the disease in the group of children fed with AAFs formulas was significantly earlier than the EHFs group, which can be justified due to the greater severity of symptoms in this group. Special feeding was started significantly earlier in this group than the other group, which was expected due to the early diagnosis of the disease in this group. According to the study of Nunes et al., if the diagnosis of food allergy is quick, the symptoms caused by the allergy will be reduced, as a result of weight loss and nutrient absorption will also be reduced [30]. Therefore, in justifying the absence of growth disorders in these infants, we can point to the quick diagnosis and early initiation of a suitable nutritional regimen.

## Conclusion

Overall, the findings of this study showed that the growth criteria (weight, length, and head circumference) in infants with CMPA in both groups fed with EHFs and AAFs formula compared to age and birth criteria were normal range. But these criteria were higher in the EHFs group compared to the AAFs group. Therefore, in infants fed with AAFs formulas, it is important to prescribe appropriate nutritional supplements to meet the child's nutritional needs.

The most important limitation of this study is its small sample size, which is due to the strict and high exclusion criteria of this study, that only healthy children without any underlying disease were included in the study. Another limitation was obtaining the correct information about the patients' history, which was used to obtain this information from the vaccine card or their medical records.

### Recommendation

It is suggested that in the future, prospective group studies with a larger sample size and a long follow-up period in each group should be conducted to investigate the effectiveness and benefit of each type of formula in terms of growth and also the return of tolerance to cow's milk protein.

### Supplementary Information

Supplementary Material 1.

## Data Availability

The datasets used and/or analyzed during the current study are available from the corresponding author on reasonable request.

## Abbreviations

AAFs: Amino acid-based formulas
ANCOVA test: Analysis of Covariance
BMI: Body mass index
CMPA: Cow’s milk protein allergy
EHFs: Extensively hydrolyzed formulas
GERD: Gastroesophageal reflux disease
WHO: World Health Organization
