# Supplementary material for: Growth in healthy infants with cow's milk protein allergy fed extensively hydrolyzed or amino acid-based formulas
Source: BMC Nutr. 2024 Jul 19;10:101. doi: 10.1186/s40795-024-00901-6 (PMC11264743; doi:10.1186/s40795-024-00901-6)
Supplement: Supplementary file 1 — Supplementary Material 1. [file 40795_2024_901_MOESM1_ESM.docx]

**Supplementary Table: Questionnaire**

Name………………………………… Surname…………………………..

Gender: Male 🖵 Female 🖵

Date of birth………………………….

National Code…………………….......

Mailing Address……………………………………………………………………………………

Email Address…………………………………………..

Phone Number……………………………………Cell Phone Number…………………………...

Time of disease diagnosis………………………………..

Time of starting special feeding………………………….

Time interval between diagnosis and treatment……………………..

Clinical manifestations:

- Gastroesophageal reflux 🖵
- Dysentery 🖵
- Eczema 🖵
- Vomiting 🖵
- Cough 🖵
- Other 🖵

Birth order: First 🖵 Second 🖵 Third 🖵 Other 🖵

History of allergy: Yes 🖵 No 🖵

Family history of allergy: Yes 🖵 No 🖵

Mother’s education level: Diploma 🖵 Associate degree 🖵 Bachelor's 🖵 Master’s 🖵 doctor 🖵

Type of hypoallergenic formula:

- Extensively hydrolyzed (EHFs) formula 🖵
- Amino acid-based (AAFs) formula 🖵

| Growth criteria | At birth | Two months | Four months | Six months | One year |
| --- | --- | --- | --- | --- | --- |
| Weight (gr) |  |  |  |  |  |
| Height (cm) |  |  |  |  |  |
| Head circumference (cm) |  |  |  |  |  |
| BMI |  |  |  |  |  |
